# Co-Immobilization of *Rhizopus oryzae* and *Candida rugosa* Lipases onto mMWCNTs@4-arm-PEG-NH_2_—A Novel Magnetic Nanotube–Polyethylene Glycol Amine Composite—And Its Applications for Biodiesel Production

**DOI:** 10.3390/ijms222111956

**Published:** 2021-11-04

**Authors:** Saadiah A. Abdulmalek, Kai Li, Jianhua Wang, Michael Kidane Ghide, Yunjun Yan

**Affiliations:** 1 Key Laboratory of Molecular Biophysics of the Ministry of Education, College of Life Science and Technology, Huazhong University of Science and Technology, Wuhan 430074, China; micro.saadiah2015@hotmail.com (S.A.A.); 2019506019@hust.edu.cn (K.L.); jhwang@hust.edu.cn (J.W.); michaelk438@gmail.com (M.K.G.); 2 Department of Biology, Faculty of Science, Sana’a University, Sana’a 1247, Yemen; 3 Department of Biology, Eritrea Institute of Technology, Mainefhi College of Science, Mainefhi 12676, Eritrea

**Keywords:** biodiesel, co-immobilization, 4-arm-PEG-NH_2_, lipase, ultrasound-assisted enzymatic reaction

## Abstract

This article describes the successful synthesis of a novel nanocomposite of superparamagnetic multi-walled nanotubes with a four-arm polyethylene glycol amine polymer (mMWCNTs@4-arm-PEG-NH_2_). This composite was then employed as a support for the covalent co-immobilization of *Rhizopus oryzae* and *Candida rugosa* lipases under appropriate conditions. The co-immobilized lipases (CIL-mMWCNTs@4-arm-PEG-NH_2_) exhibited maximum specific activity of 99.626U/mg protein, which was 34.5-fold superior to that of free ROL, and its thermal stability was greatly improved. Most significantly, CIL-mMWCNTs@4-arm-PEG-NH_2_ was used to prepare biodiesel from waste cooking oil under ultrasound conditions, and within 120 min, the biodiesel conversion rate reached 97.64%. This was due to the synergy effect between ROL and CRL and the ultrasound-assisted enzymatic process, resulting in an increased biodiesel yield in a short reaction time. Moreover, after ten reuse cycles, the co-immobilized lipases still retained a biodiesel yield of over 78.55%, exhibiting excellent operational stability that is attractive for practical applications. Consequently, the combined use of a novel designed carrier, the co-immobilized lipases with synergy effect, and the ultrasound-assisted enzymatic reaction exhibited potential prospects for future applications in biodiesel production and various industrial applications.

## 1. Introduction

Recently, biodiesel has received increased attention due to its biodegradable, renewable, and non-toxic properties that offer significant environmental and economic benefits [1], which provide an eco-friendly solution to fuel crisis [2]. It is a chemical and technological bioenergy liquid [3] composed of a mixture of fatty acid alkyl esters derived from a variety of oil sources, including animal fats, vegetable oils [4], and algal and microbial oils [5]. The oil interacts with alcohol via transesterification in the existence of lipases or chemical catalysts to produce biodiesel [6]. Ardabili et al. [7] produced biodiesel from waste cooking oil (WCO) using NaOH as a chemical catalyst by applying an extreme learning machine (ELM) and response surface methodology (RSM). In another study by Najafi et al. [8], the adaptive neuro-fuzzy inference system (ANFIS), artificial neural networks (ANNs), and RSM were employed to produce biodiesel from WCO using chemical catalysts. Based on the results, the operation to produce biodiesel by chemical catalysts is faster than lipase as the catalyst. Compared to chemical catalysts, lipases are biochemical green catalysts that are preferred in transesterification reactions because they use lower energy, require simpler operating conditions, generate higher purity products, and can be simply recovered. Moreover, lipases have a broad substrates specificity [9,10].

Lipases (triacylglycerol acyl hydrolase EC 3.1.1.3) are the most promising industrial enzymes for biodiesel synthesis [9]. The specificity of lipases is a significant factor for their industrial applications [11]. Depending on this feature, lipases can be categorized into three types: sn-1,3-specificity, sn-2-specificity, and non-specificity [12]. However, multiple lipases with different specificity sites (nonspecific lipase and sn-1,3-specific lipase) could be employed concurrently to avoid acyl movement as a rate-limiting step in biodiesel production, to eliminate the accumulation of intermediate products, to reduce the reaction time, and to enhance lipase activity and biodiesel yield [13].

On the other hand, the industrialization of biodiesel catalyzed by lipase has been affected by long reaction times and inefficient production [12]. Maximum yields of biodiesel could be obtained in a short reaction time via combining two or more lipases with different sites specificity rather than a single lipase [12,14,15,16], as the combined lipases will act synergistically, targeting various triglycerides sites in the oil composition [17], and avoiding the formation of 2-monoglycerides during the reaction [13]. In addition, the use of more than one lipase may reduce biocatalyst costs due to their different market prices [18]. From the same point, the highest yield of biodiesel could be achieved in a short reaction time by applying an ultrasonic process in an enzymatic transesterification reaction [19,20]. Ultrasound-assisted technology has been reported as a promising technique because it can overcome the reactants’ low miscibility in a transesterification reaction; thereby, improving mass transfer and reaction rates, and reducing reaction times [19]. The combination of a combined lipase and ultrasound has demonstrated significant innovation potential. Numerous studies should be conducted to optimize the use of this combined technology for biodiesel production [21].

However, the industrial applications of lipases encounter several obstacles, like the relatively higher cost of lipase, its low stability, and its easy inactivation by chemical or physical agents [6]. These obstacles render the effectiveness of biodiesel production unattractive, when considered economically [22]. The use of appropriate enzymatic immobilization techniques can effectively enhance thermal and chemical stabilities and reusability of lipases, protect lipase from denaturation, and reduce the costs associated with enzyme use, as well as allowing lipases to be industrially applicable [3].

Various immobilization techniques, such as co-immobilized lipases (CIL) [13,22,23,24] and a mixture of immobilized lipases (MIL) [12,25], have been used to enhance biodiesel production rates. From an environmental and economic standpoint, co-immobilization of multi-enzymes is a powerful method with numerous advantages, such as avoiding mass transfer restrictions, eliminating product contamination, preventing inactivation by intermediate products, and increasing the reaction speed [14]. The primary objective of co-immobilization was to overcome the mass transfer restrictions imposed by reactants and to enhance the reaction rate and speed [13]. There are a few previous studies using co-immobilized lipases for biodiesel production. Among them, Lee et al. reported the simultaneous immobilization of *Candida rugosa* lipase and *Rhizopus oryzae* lipase onto silica gel [22] and silanizated activated carbon [23]. They confirmed that there is a synergetic effect between CRL and ROL, and that the combination enzymes produced higher yields of biodiesel than either CRL or ROL alone.

In addition, in our previous work, the mixtures of synergetic liquid [26], immobilized lipases [12], and commercial lipases [18] were investigated to enhance biodiesel production. Moreover, in the last work [27], a process of biodiesel production, catalyzed by synergetic co-displayed ROL and CRL-1, on the cell surface of *Phichia pastoris*, was studied. All the results demonstrated that the biodiesel yield improved when a mixture of synergetic lipases was used.

There are many methods for enzymatic immobilization, such as covalent bonding, cross-linking, adsorption, and encapsulation methods [28]. Among these methods, covalent bonding of the enzyme onto the support is the most practical method. It has more attention due to the firm interaction between the enzyme and the support materials, inhibiting the enzyme leakage from the support and increasing enzyme stability [13,28,29]. To carry out lipase immobilization, nano-scale magnetic carbon nanotubes (mCNTs) have been widely investigated by researchers. These materials have a high specific surface area, wide pores and cavities in which the enzymes can be housed, own good heat conductivity, possess efficient chemical stability, and maintain easy magnetic separation [29]. However, m-CNTs lack sufficient sites for lipase immobilization, which makes them restricted in practical applications. In this regard, functionalizing their surface with dendrimers, functional materials, or hyper-branched polymers can increase enzyme loading and stability. Recently, dendrimers have been widely studied by previous researchers in the field of CNTs modification and enzyme immobilization [29]. Unfortunately, they are time consuming and use high volumes of chemicals, making the enzyme catalytic reactions high cost, and some chains of dendrimers tend to curl back on themselves randomly, resulting in a lower loading of enzymes [29,30]. Hence, it is an urgent and critical task to develop novel proper approaches to functionalize the surfaces of CNTs for the immobilization of more than one lipase and to improve biodiesel yield.

Hyperbranched polymers, such as multi-arm polyethylene glycol amine (PEG), containing a large number of amino groups, can be employed to fabricate the surface of magnetic nanomaterials and increase their capacity for enzyme loading. It can also increase enzyme catalytic activity and make the catalytic reactions of enzymes low-cost [31,32,33]. This polymer is highly water-soluble and biocompatible, enabling it to enhance the carrier’s water-soluble and biocompatible properties and protect the carrier structure, resulting in increased immobilized enzyme activity and stability [34].

Four-arm polyethylene glycol amine (4-arm-PEG-NH_2_) is a type of polyethylene glycol derivative useful for nanomaterials’ surface modification [35]. This compound contains many amino groups, and can, thus, create an immobilized enzyme when glutaraldehyde (a cross-linking agent) is bound with protein, increasing the enzyme’s stability and loading capacity. Additionally, 4-arm-PEG-NH_2_ can create a stable amide bond with the carboxyl group of materials, enhancing their solubility and stability [33]. To our knowledge, modification of magnetic MWCNTs by 4-arm-PEG-NH_2_ polymer, and their application for co-immobilization lipases have not been reported previously.

Thus, in this work, the surfaces of mMWCNTs were modified with 4-arm-PEG-NH_2_ to create a simple novel biocompatible polymer composite of magnetic nanotubes (mMWCNTs@4-arm-PEG-NH_2_), which was then applied to covalently co-immobilize two different types of specificity lipases, namely *Rhizopus oryzae* lipase (ROL) (a 1,3-specific lipase) and *Candida rugosa* lipase (CRL) (a nonspecific lipase). This newly fabricated carrier possesses several advantageous properties for lipase immobilization. Its magnetic properties enable it to be easily separated from the reaction, and it is characterized by a large surface area and a high number of amino groups on its surface, which improves lipase loading and stability. Then, the morphological characteristics of the fabricated samples were examined. The effects of various co-immobilization conditions were further investigated. Following that, in a co-solvent system, the co-immobilized lipase (CIL-mMWCNTs@4-arm-PEG-NH_2_) was employed to catalyze the transesterification reaction of waste cooking oil with the assistance of an ultrasound technique to prepare biodiesel. Furthermore, different transesterification parameters were optimized to enhance the biodiesel yields.

The main purpose of this study was employed to use the co-immobilized lipases–mMWCNTs@4-arm-PEG-NH_2_ as a biocatalyst to produce biodiesel, WCO as a feed-stock, and the ultrasonic technique as an assisted technique in the transesterification reaction, to produce a high yield of biodiesel in the short reaction time and reduce the production cost, which is one of the limitations in the biodiesel production process. This designed method showed better biodiesel production than that of previously reported immobilized lipases on functionalized MWCNTs [36].

## 2. Results and Discussion

### 2.1. Design and Characterization of CIL-mMWCNTs@4-arm-PEG-NH_2_

**Scheme 1:**
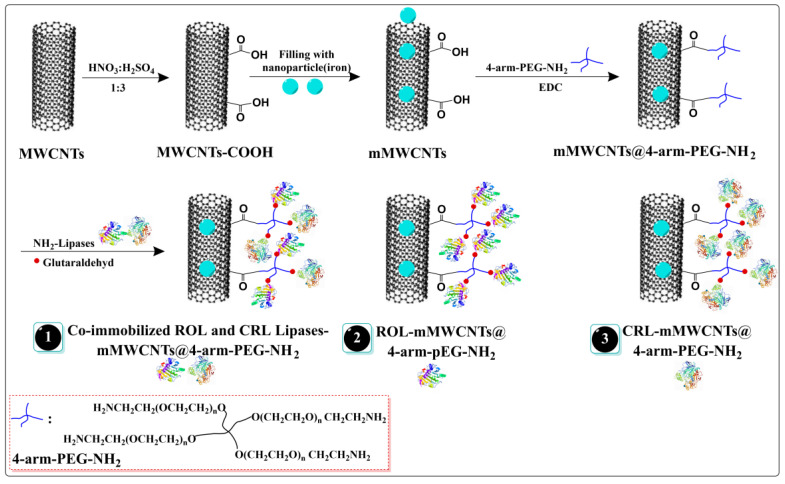
Diagram for the synthesis of mMWCNTs@4-arm-PEG-NH_2_ and their application in lipase immobilization.

A schematic diagram for the fabrication of mMWCNTs with a hyperbranched 4-arm-PEG-NH_2_ polymer (mMWCNTs@4-arm-PEG-NH_2_), and subsequently their coupling with ROL and CRL enzymes, is depicted in Scheme 1. Firstly, crude MWCNTs were oxidized to form MWCNTs-COOH. Next, the magnetic carbon nanotubes (mMWCNTs-COOH) were synthesized using a simple method that encapsulated Fe_3_O_4_ nanoparticles within the MWCNTs-COOH interiors. Then, following the procedure of Rong et al. [33], the 4-arm-PEG-NH_2_ polymers were grafted onto mMWCNT’s surfaces by creating amido bonds via the carboxyl–amino group reaction. Following that, glutaraldehyde was employed to link the amino groups of the support material and the lipases, resulting in the formation of immobilized lipases.

**Figure 1:**
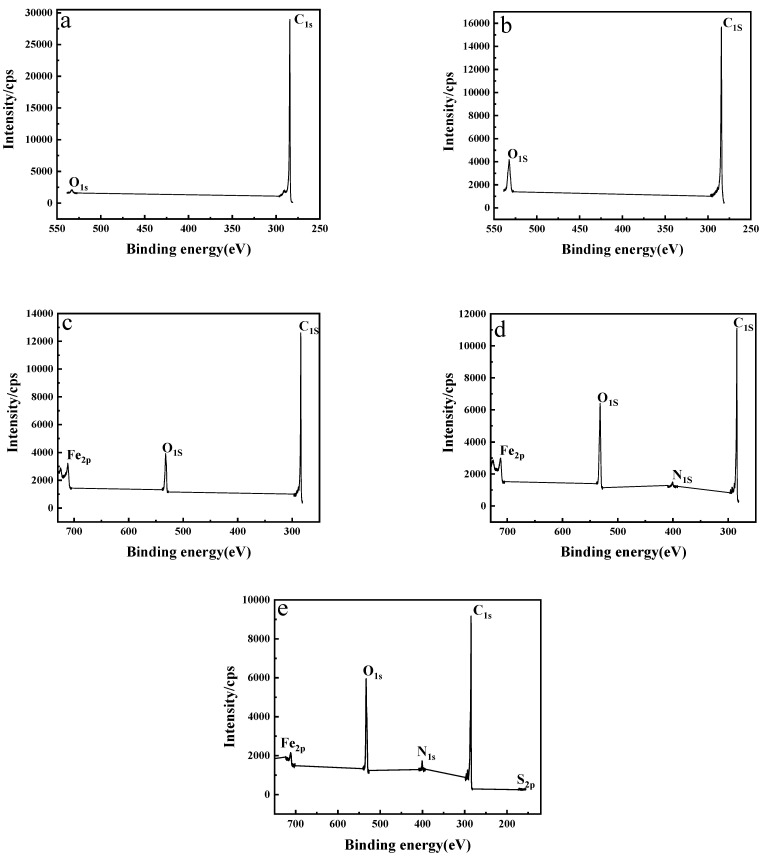
XPS spectra of (**a**) crude MWCNTs; (**b**) MWCNTs-COOH; (**c**) mMWCNTs; (**d**) mMWCNTs@4-armPEG-NH_2_; (**e**) co-immobilized ROL and CRL-mMWCNTs@4-armPEG-NH_2_.

XPS was used to investigate all the above processes (Figure 1). The surface composition of the MWCNTs was determined using XPS wide energy survey scans. C_1s_ and O_1s_ peaks were detected in all spectra (Figure 1a–e), but their peak areas varied. Nevertheless, the treated MWCNTs had a higher oxygen content than the crude MWCNTs, so the O_1s_ signal was different. The Fe_2p_ peak in Figure 1c revealed that MWCNTs with iron particles were generated, which came from Fe_3_O_4_ nanoparticles located either inside or outside the MWCNTs-COOH. The spectrum contained prominent peaks of Fe_2p_ were (711.05), O_1s_ (531), and C_1s_ (284) (Table 1). After fabrication of mMWCTs with 4-arm-PEG-NH_2_, a peak matching to N_1s_ (399.70) was introduced to the carriers (Figure 1d and Table 1), confirming the successful modification of mMWCTs with 4-arm-PEG-NH_2_. When lipase was immobilized onto the carrier, an S_2p_ peak was observed on the carrier’s surface, which was attributable to the lipase’s cysteine (Table 1). The difference in elementary contents and kinds is a clear indicator for successful modification of MWCNTs and co-immobilization, as N was absent in crude MWCNTs, MWCNTs-COOH, and mMWCNTs, and S existed only in lipases. It can be seen from Figure 1 and Table 1 that MWCNTs were successfully modified to form MWCNTs-COOH, mMWCNTs, mMWCNTs@4-arm-PEG-NH_2_, and lipases–mMWCNTs@4-arm PEG-NH_2_. 

**Figure 2:**
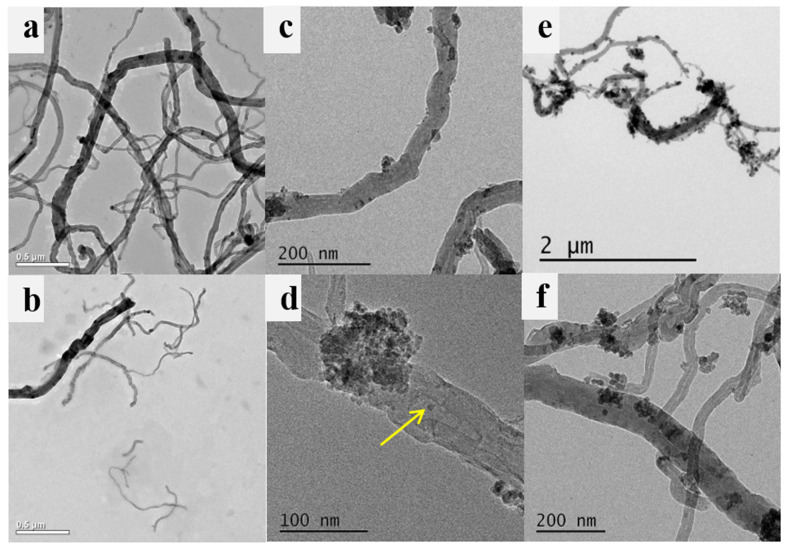
TEM images of (**a**) crude MWCNTs; (**b**) oxidized MWCNTs; (**c**) magnetic MWCNTs; (**d**) magnified image of mMWCNTs with iron oxide nanoparticles in its internal cavity; (**e**,**f**) mMWCNTs modified with 4-arm-PEG-NH_2_ polymer.

**Figure 3:**
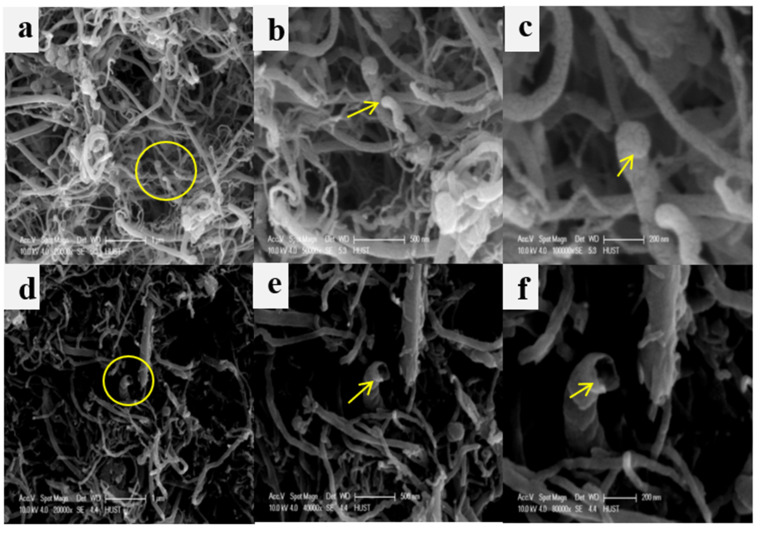
SEM images of MWCNTs before and after oxidation at different magnification scales: (**a**–**c**) crude MWCNTs; (**d**–**f**) oxidized MWCNTs.

The TEM technique was used to observe the MWCNTs’ morphological alterations. The analysis was performed on crude MWCNTs, oxidized MWCNTs, mMWCNTs, and mMWCNTs@4-arm-PEG-NH_2_ (Figure 2). As observed in Figure 2a,b, the TEM images of crude and oxidized MWCNTs, prior to the modification process, are free of particle-like characteristics, and the oxidized MWCNTs are shorter than the non-oxidized MWCNTs. Following magnetic treatment, MWCNTs contain Fe_3_O_4_ nanoparticles inside their internal cavity, while a few nanoparticles are deposited randomly on the MWCNTs’ external surfaces (Figure 2c,d). TEM images of mMWCNTs, modified with a 4-arm-PEG-NH_2_ polymer, are shown in Figure 2e,f. Compared with the crude MWCNTs, oxidized MWCNTs, and mMWCNTs without 4-arm-PEG-NH_2_, the surface of mMWCNT@4-arm-PEG-NH_2_ has become roughened, owing to the attachment of hyperbranched molecules to the carboxylated magnetic nanotubes. This clearly shows the successful modification of 4-arm-PEG-NH_2_ onto mMWCNTs surface. Under FSEM, crude and oxidized MWCNTs were also examined to check the surface morphology characteristics of MWCNTs before and after oxidation (Figure 3). As shown from Figure 3a–c, the crude MWCNTs appeared as bundles, and their ends were closed before oxidation. In comparison, the tubes seemed to be shorter in length, and their ends were opened after oxidation treatments (Figure 3d–f). The carbon–carbon bond was broken during acid oxidation of nanotubes, oxygen units were introduced onto the nanotubes’ surfaces, and the oxidizing agent was diffused through the nanotubes, resulting in the elimination of impurities, the consumption of carbon, and the creation of pores. Thus, this process formatted the pores and increased the number of available termini to connect further functional groups, which improves MWCNT dispersion in water and organic solvents and enables additional modification of MWCNTs [37].

**Figure 4:**
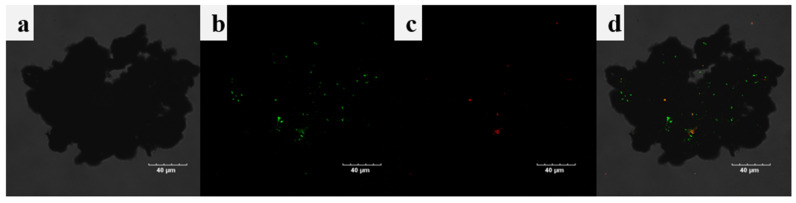
CLSM micrographs of the co-immobilized lipases onto mMWCNTs@4-arm-PEG-NH_2_; (**a**) bright-field micrograph; (**b**) dark field of RhB-labeled CRL fluorescence micrograph (**c**); dark field of FITC-labeled ROL fluorescence micrograph (**d**); overlay of a bright field, RhB-labeled CRL and FITC-labeled ROL fluorescence micrograph.

To further demonstrate the success of co-immobilization, CLSM was employed to provide visual evidence, which directly confirmed the presence of targeted ROL and CRL molecules on the prepared mMWCNTs@4-arm-PEG-NH_2_ (Figure 4a–d). Prior to co-immobilization, ROL and CRL were pre-treated with fluorescein isothiocyanate (FITC) and rhodamine B (RhB), respectively. The CLSM micrograph demonstrated that the fluorescein iso-thiocyanate (FITC)-labeled ROL and the rhodamine B (RhB)-labeled CRL were co-immobilized onto the mMWCNTs@4-arm-PEG-NH_2_. The bright micrograph in Figure 4a was acquired using an inverted phase-contrast microscope. The green, fluorescent signal on the micrograph was obtained from the ROL tagged with fluorescein iso-thiocyanate (FITC) (Figure 4b), and the red fluorescent signal on the micrograph was derived from CRL tagged with rhodamine B (Figure 4c). The presence of the green and red fluorescent signals at the overlay of a bright field, the FITC-labeled ROL fluorescence micrograph, and RhB-labeled CRL fluorescence micrograph (Figure 4d) confirms successful co-immobilization of ROL and CRL onto the mMWCNTs@4-arm-PEG-NH_2_ support.

**Figure 5:**
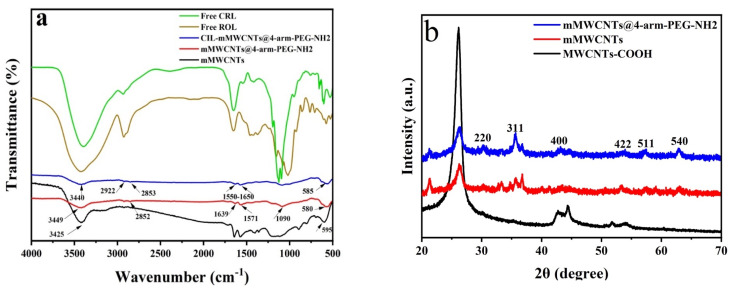
(**a**) FT-IR spectra of MWCNTs; (**b**) XRD patterns of MWCNTs.

Figure 5a shows the FT-IR spectra of the synthesized samples. As shown, mMWCNTs, mMWCNTs@4-arm-PEG-NH_2_, and CIL-mMWCNTs@4-arm-PEG-NH_2_ spectra were exhibited distinctly strong absorption bands at the peaks 595, 580, and 585 cm^−1^, respectively, attributed to the Fe–O bond from Fe_3_O_4_. Bands at 1090, 1571, and 2852 cm^−1^ in the mMWCNTs@4-arm-PEG-NH_2_ spectrum were corresponding to C-O, N-H, and C-H vibrations, respectively, proving that 4-arm-PEG-NH_2_ had been grafted onto mMWCNTs, respectively [33]. Moreover, additional bands were observed in the CIL-mMWCNTs@4-arm-PEG-NH_2_ spectrum at peaks 2853 and 2922 cm^−1^, dedicated to C-H stretching vibrations of the glutaraldehyde and lipases [38]. The absorption bands detected between 1550 and 1650 cm^−1^ peaks are characteristics of the amide I band, (C=O stretching, 1650 cm^−1^) and amide II band (N-H bending, 1550 cm^−1^) vibrations of the protein [39], indicating that the lipases were successfully co-immobilized on the support. Additionally, there are additional peaks on the supports between 3420 and 3450 cm^−1^ that could be attributed to amino acid −OH vibrations [39].

In addition, the crystalline structure of Fe_3_O_4_, in synthetic magnetic nanotubes, was determined using XRD. The XRD patterns of the MWCNTs-COOH, mMWCNTs, and mMWCNTs@4-arm-PEG-NH_2_ were collected (Figure 5b). As illustrated in Figure 4b, the diffraction pattern of mMWCNTs and mMWCNTs@4-arm-PEG-NH_2_ contained six significant characteristic peaks localized at 2θ = 30.22°, 35.59°, 43.32°, 53.37°, 57.13°, and 62.82°, which correspond to the (220), (311), (400), (422), (511), and (440) of cubic Fe_3_O_4_ (JCPDS file no. 85-1436), respectively. The distinctive peaks of mMWCNTs remained rather stable after modification with 4-arm-PEG-NH_2_ polymers, indicating that modification did not have an impact on the crystal construction of Fe_3_O_4_ nanoparticles. In comparison, the XRD pattern of MWCNTs-COOH lacks these six peaks.

**Figure 6:**
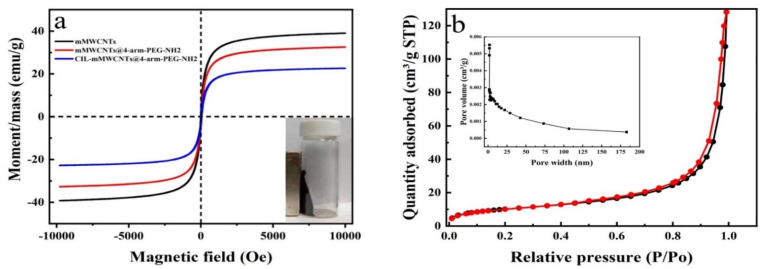
Magnetic characteristics, surface area, and pore size of mMWCNTs support. (**a**) Magnetic hysteresis curves of mMWCNTs samples; the insert photographic image shows CIL-mMWCNTs@4-arm-PEG-NH_2_ attracted by an external magnet. (**b**) Surface area and pore size analysis for mMWCNTs@4-arm-PEG-NH_2_.

Moreover, the magnetic characteristics of mMWCNTs, mMWCNTs@ 4-arm-PEG-NH_2_, and CIL-mMWCNTs@4-arm-PEG-NH_2_ were investigated by VSM analysis (Figure 6a). As illustrated in Figure 6a, the saturation magnetization of mMWCNTs, mMWCNTs@4-arm-PEG-NH_2_, and CIL-mMWCNTs@4-arm-PEG-NH_2_ was 39.03, 32.53, and 22.63 emu/g, respectively. Due to the presence of proteins, the saturation magnetization of CIL-mMWCNTs@4-arm-PEG-NH_2_ was lower than that of mMWCNTs@4-arm-PEG-NH_2_. In general, the magnetization curves of all tested samples showed no hysteresis, which demonstrated their superparamagnetic property. Therefore, the magnetic samples could be rapidly separated from the reaction mixture using an external magnet. Once the magnetic field is released, mechanical shaking can be easily dispersed the immobilized enzyme in the reaction mixture.

Additionally, Figure 6b illustrates the N_2_ adsorption–desorption isotherm mMWCNts@4-arm-PEG-NH_2_, using BET analysis. The surface area of mMWCNTs@4-arm-PEG-NH_2_ reached to 37.45 m^2^/g, with an average pore width of 21.17 nm: these results demonstrate that the nanomaterial is mesoporous. Remarkably, the large pore size allows a decline in the mass transfer resistance of biomacromolecules after the immobilization process [30], indicating that the designed mMWCNTs@4-arm-PEG-NH_2_ is an excellent tool for enzyme immobilization.

In order to collect excellent characteristics (such as simplicity of separation and recovery, and suitable active sites) onto a single support for enzyme immobilization, in this study, hyperbranched-functionalized CNTs, encapsulated with magnetic Fe_3_O_4_ nanoparticles, were prepared. The characterization results indicated that the surface of mMWCNTs provided sufficient active sites for co-immobilization of two lipases, following the grafting process. The mMWCNTs@4-arm-PEG-NH_2_ exhibited a rapid response to an externally applied magnetic field, indicating that this degree of saturation magnetization is sufficient for immobilized enzyme separation.

### 2.2. Co-Immobilization of ROL and CRL onto mMWCNTs@4-armPEG-NH_2_

Restriction of two or more enzymes at the same support is known as co-immobilization [40]. It is a highly effective technique for increasing the enzyme’s stability and reusability [14]. Generally, the immobilization parameters significantly affect the immobilization efficiency and specific activity, including the ratio of lipases, the concentration of glutaraldehyde, the pH value, the immobilization temperature, and the immobilization time; thus, they were all examined in this work.

**Figure 7:**
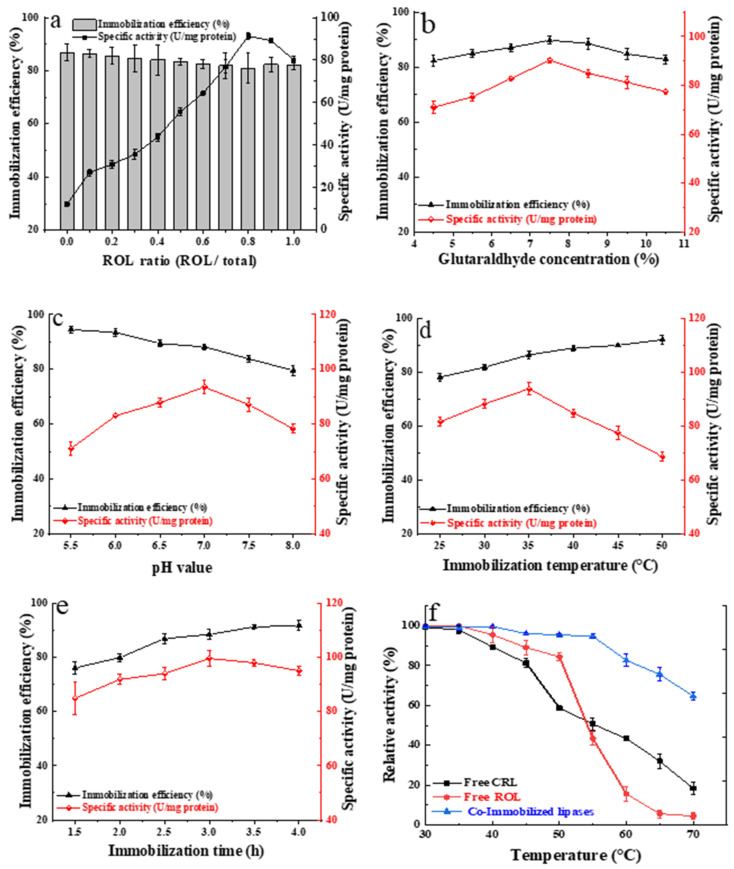
Effects of immobilization conditions on the immobilization efficiency and specific activity of CIL-mMWCNTs@ 4-arm-PEG-NH_2_: (**a**) ROL ratio; (**b**) glutaraldehyde concentration; (**c**) pH value; (**d**) immobilization temperature; (**e**) immobilization time; (**f**) thermo-stability of co-immobilized lipases. The optimal immobilization conditions were 4:1 ROL: CRL ratio; 7.5 wt.% glutaraldehyde concentration; 7.0 pH value; 35 °C immobilization temperature; 3 h immobilization time.

Initially, the synergistic effect between ROL and CRL on co-immobilization efficiency and specific activity was investigated. The enzymes were covalently coupled in various ratios (0.0–1.0) onto mMWCNTs@4-arm-PEG-NH_2_ (Figure 7a), and the initial protein content was identical in all ratios. Results showed that the specific activity exhibited a rapid increase with increasing ROL dosage up to 0.8 ratio, followed by a decline. When the ROL ratio was 0.8, the maximum specific activity was observed (91.346 U/mg protein), indicating that the optimum ratio of ROL to CRL was 4:1. The specific activity of this ratio was higher than the specific activity of ROL and CRL when used alone. These findings are in contract with the earlier study reported by Lee et al. [22]. They proved that the specific activity of the co-immobilized lipases was more significant than that of a single immobilized lipase, which means that the mass transfer between ROL and CRL enzymes was significantly improved during the reaction. On the other hand, the immobilization efficiency was not obviously changed at various ratios. Hence, 0.8 of ROL ratio was selected as the optimum lipases’ ratio for further investigation.

The amino groups of ROL and CRL enzymes and mMWCNTs@4-arm-PEG-NH_2_ can be ligated by glutaraldehyde. Although glutaraldehyde is a non-toxic cross-linker, its high concentration can inactivate the immobilized enzyme due to glutaraldehyde polymerization or intermolecular enzyme cross-linking, preventing substrate access to the enzyme catalytic site [29]. Simultaneously, at too low concentrations of glutaraldehyde, the amino groups in mMWCNTs@4-arm-PEG-NH_2_ are insufficiently activated, resulting in the leakage of unbound ROL and CRL enzymes into the aqueous medium. Therefore, the suitable concentration of glutaraldehyde should be determined. As seen in Figure 7b, the CIL-mMWCNTs@ 4-arm-PEG-NH_2_ showed reduced in immobilization efficiency and specific activity at two high and low glutaraldehyde concentrations. The optimal concentration of glutaraldehyde for cross-linking was determined to be 7.5 wt.%. At this concentration, the specific activity of the co-immobilized lipases reached its maximum value (90.278 U/mg protein). This was similar with Fan et al. [36] who got a maximal specific activity at glutaraldehyde concentration (7.5%) for three different types of immobilized lipases (*Burkholderia cepacia* lipase, *Rhizomucor miehei* lipase, and CRL). All three lipases were covalently immobilized onto dendrimer-functionalized mMWCNTs, and they demonstrated that the same quantity of glutaraldehyde was needed to fully activate the amino group of the identical carrier. Lee et al. [23] applied functionalized activated carbon for co-immobilized ROL and CRL and obtained the highest specific activity when the glutaraldehyde concentration was 8%.

In addition, pH value has a significant impact on the enzyme’s co-immobilization process (Figure 7c). As shown, a neutral medium is beneficial for co-immobilizing ROL and CRL onto mMWCNTs@4-arm-PEG-NH_2_, which is consistent with previous reports by Li et al. [38]. A remarkable increase of the specific activity from 71.047 to 93.483 U/mg protein was obtained when a pH value increased from 5.5 to 7.0. However, as the pH value increased above 7.0, the specific activity and immobilization efficiency both decreased, indicating that the immobilized enzyme is less stable at a higher pH value. Based on the above results, pH 7.0 was selected as the optimal value for co-immobilization of ROL and CRL.

In Figure 7d, the effects of various immobilization temperatures (25–50 °C) on the immobilization efficiency and specific activity were investigated. The results indicated that the immobilization efficiency was significantly increased from 78.32% to 92.28% as the immobilization temperature increased from 25 °C to 50 °C, evidencing that the covalent co-immobilization is an endothermic reaction. In contrast, the highest specific activity of 94.017 U/mg (ROL and CRL) was obtained at 35 °C. Beyond 35 °C, the specific activity started to decline due to the thermal denaturation of protein. Depending on the immobilization kinetics, the reaction temperature can enhance the binding of the carrier to the lipase and lead to improving the immobilization efficiency [9,38]. However, when the reaction temperature rises further, the activity of the lipases will be decreased due to the changes in their molecular conformation, resulting in a decline in the co-immobilized lipases activity [6]. For this, a co-immobilization temperature of 35 °C was regarded as the ideal option for further study.

Subsequently, ROL and CRL were covalently co-immobilized on mMWCNTs@4-arm-PEG-NH_2_ at various immobilization times (Figure 7e). As seen in Figure 7e, the immobilization efficiency and lipases activity were increased with the immobilization time increased, which reached the highest value of 99.626 U/mg after 3 h. However, the specific activity began to decline when the immobilization time exceeded 3 h, this could be because the covalent interaction between the amino groups of mMWCNTs@4-arm-PEG-NH_2_ and the enzymes increases, forming an intermolecular steric barrier between the lipase molecules [30]. Hence, the optimum immobilization time was set at 3 h. This was in agreement with previously reported findings [30]. Furthermore, Lee et al. [23] reported the simultaneous immobilization of CRL and ROL onto functionalized activated carbon. They showed that the co-immobilized lipases had the highest activity after 24 h of immobilization reaction time.

In addition, thermal stability is critical for the practical applications of enzymes. The thermal stabilities of CIL-mMWCNTs@4-arm-PEG-NH_2_, free ROL, and free CRL were depicted in Figure 7f. The results indicated that, when the incubation temperature was increased from 30 °C to 70 °C, the relative activities of all lipases were decreased. The decline in free lipases activities was significantly greater than that of the immobilized form. Furthermore, co-immobilization obviously improved the thermal stability of ROL and CRL. When the temperature range was at 70 °C, the relative activity of the CIL-mMWCNTs@4-arm-PEG-NH_2_ was greater than 60%. These findings demonstrated that the covalent bonding of ROL and CRL onto the support can protect lipase molecules from thermal denaturation. Therefore, the conformation of ROL and CRL onto a novel mMWCNTs@4-arm-PEG-NH_2_ polymer was more stable, resulting in a wide tolerance to temperature.

Under optimal immobilization conditions, the specific activity of CIL-mMWCNTs@4-arm-PEG-NH_2_ was 34.5-fold and 6.2-fold more significant than that of free ROL and CRL, respectively. Additionally, after co-immobilization, the thermal stability of ROL and CRL was significantly enhanced. Numerous factors contributed to these significant properties of the co-immobilized lipases. The first one is associated with the immobilization method. Covalent bonding is a chemical immobilization method between the lipase and a novel designed hyperbranched mMWCNTs carrier. This covalent bond that formed between the enzyme and the carrier is extremely strong and stable compared to other immobilization methods [36,38]. In comparison with other previous covalent methods, this is a simple and effective method that overcomes their disadvantages, including a lengthy time, high temperature, and solvents that can affect enzymes’ structure, activity, and stability. Second, the co-immobilization technique of enzymes is a technique with many benefits. The mixture of a 1,3-specific lipase and a nonspecific lipase, co-immobilizing them onto the same support, can improve the synergistic effect between lipases, decrease the mass transfer, eliminate the reaction by-products, and increase the enzyme activity [14,22,23]. The third factor is related to the carrier properties—as explained previously, hyperbranched polymer fabricated nano-scale materials have unique properties. They can increase surface area and the contact area between the enzyme and the substrate, which effectively results in higher immobilization efficiency and decreases mass transfer resistance, resulting in enhanced enzyme activity [33,34,41]. The final factor is related to lipases. The majority of lipases include a portable component referred to as the “lid”, which surrounds the active catalytic centers and regulates substrate access to the enzyme’s active site. The secondary structure of ROL and CRL lipases is likely to change during the immobilization process. The “lid” is opened for a period to facilitate substrate access, resulting in an increase in lipase activity [42,43].

### 2.3. Transesterification of WCO for Biodiesel Production Catalyzed by Single and Co-Immobilized Lipases onto mMWCNTs@4-arm-PEG-NH_2_

Recently, the production of biodiesel, catalyzed by a combination of enzymes with varying degrees of specificity, has received more attention [16]. There are two significant drawbacks in biodiesel production, which are indicated by the high value of refined oils (as a substrate) and the long reaction time. Thus, using WCO as a biodiesel feedstock can reduce production costs, while also making the process very environmentally friendly [6].

Furthermore, the application of ultrasound in enzymatic catalytic reactions and the combination of different substrate enzymes could reduce the reaction time [12,44]. Hence, in the current study, ultrasonic technology was applied to transesterify WCO, catalyzed by a combination of two immobilized lipases, namely ROL (1,3 specific lipase) and CRL (nonspecific lipase). Both lipases were co-immobilized onto mMWCNTs@4-arm-PEG-NH_2_ support at a ratio of 4:1, respectively. Consequently, some significant transesterification parameters were successively optimized to improve the biodiesel yield. Before optimization, the effects of single and co-immobilized lipases were both investigated; after that, the ratio of co-solvent, methanol to oil molar ratio, reaction temperature, amount of co-solvent, reaction time, water content, and lipase dosage were systematically examined.

#### 2.3.1. Effect of Single and Co-Immobilized Lipases on the Yield of Biodiesel

**Figure 8:**
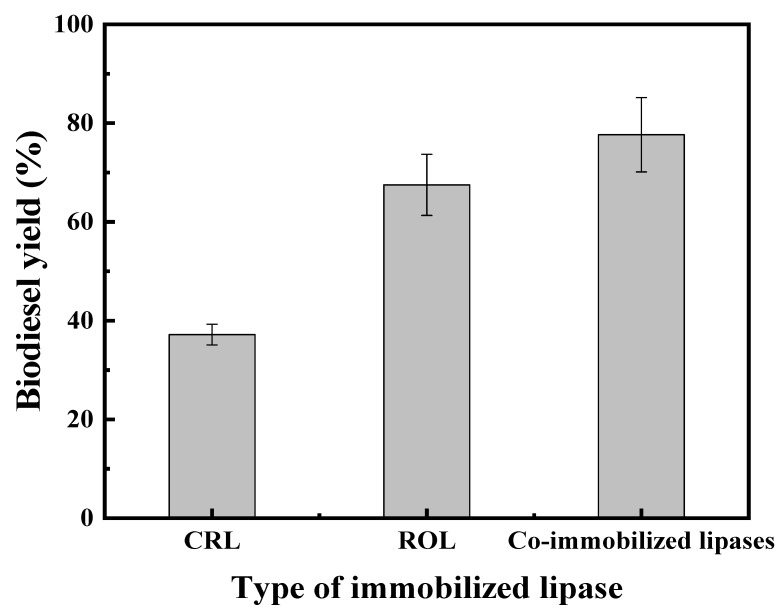
Transesterification of WCO using single and co-immobilized lipases under the following parameters: 4:1 molar ratio of methanol to oil (methanol was added 3 times at intervals of 40 min); 20% isooctane; 5% water content; 10% immobilized lipase; 40 ± 1 °C; 120 min reaction time; under ultrasound-assisted parameters of 40% power and 40 kHz frequency (all % based on oil weight).

The majority of biodiesel is generated utilizing a single lipase rather than a combination of lipases. In this study, ROL and CRL lipases were immobilized individually onto mMWCNTs@4-arm-PEG-NH_2_; furthermore, they were co-immobilized simultaneously onto mMWCNTs@4-arm-PEG-NH_2_. The effects of single and co-immobilized lipases on biodiesel yield were determined. As depicted in Figure 8, the highest biodiesel yield (77.66%) was achieved when the reaction was catalyzed by co-immobilized lipases, which is more than 10% higher than those produced by immobilized ROL or CRL alone. A single immobilized CRL showed the lowest yield of biodiesel (37.18%), followed by the immobilized ROL (67.51%). This finding indicated that the biodiesel yield catalyzed by a combination of co-immobilized lipases was significantly more than when a single immobilized lipase was used. Our findings were consistent with previous findings by Lee et al. [45], which proved that the maximum biodiesel yield of 99% was observed in 21 h, catalyzed by a combination of immobilized ROL and CRL enzymes, while the product yields of 70% and 20% were, respectively, obtained from immobilized ROL and CRL alone. Su et al. [12] found that 98.3% biodiesel yield was produced from combined immobilized lipases (Novozym 435 and ROL) at a ratio of 1:3, which resulted in a significant 30% of FAEEs conversion rate, higher than that of ROL alone. This can be explained by the transesterification mechanism, in which the immobilized lipase mixtures used to produce biodiesel depend on the lipase’s synergistic activity to breakdown the WCO content [16]. WCO was hydrolyzed to form diglycerides, monoglycerides, and free fatty acids (FFAs), and then FFAs were esterified by methanol to form methyl esters. The capacity of ROL to stimulate the sn-2 site of mono and diglycerides was limited. The addition of CRL with ROL to the reaction enhances the release rate of FFAs at the sn-2 site. Thus, it is thought that CRL assisted in the breakdown of WCO to release FFAs at sn-2 site, and then ROL esterifies them to synthesize methyl esters [12]. As a result, co-immobilization has been shown to have a significant impact on biodiesel production; thus, it can be regarded as an appropriate choice for enhancing biodiesel production. As far as we know, this is the first report of biodiesel manufacturing under ultrasound-assisted conditions using co-immobilized ROL and CRL onto mMWCNTs@4-arm-PEG-NH_2_ from WCO. Since the co-immobilized lipases improved biodiesel production yield, they were chosen for the experiments.

#### 2.3.2. Effect of Different Transesterification Parameters on the Yield of Biodiesel

**Figure 9:**
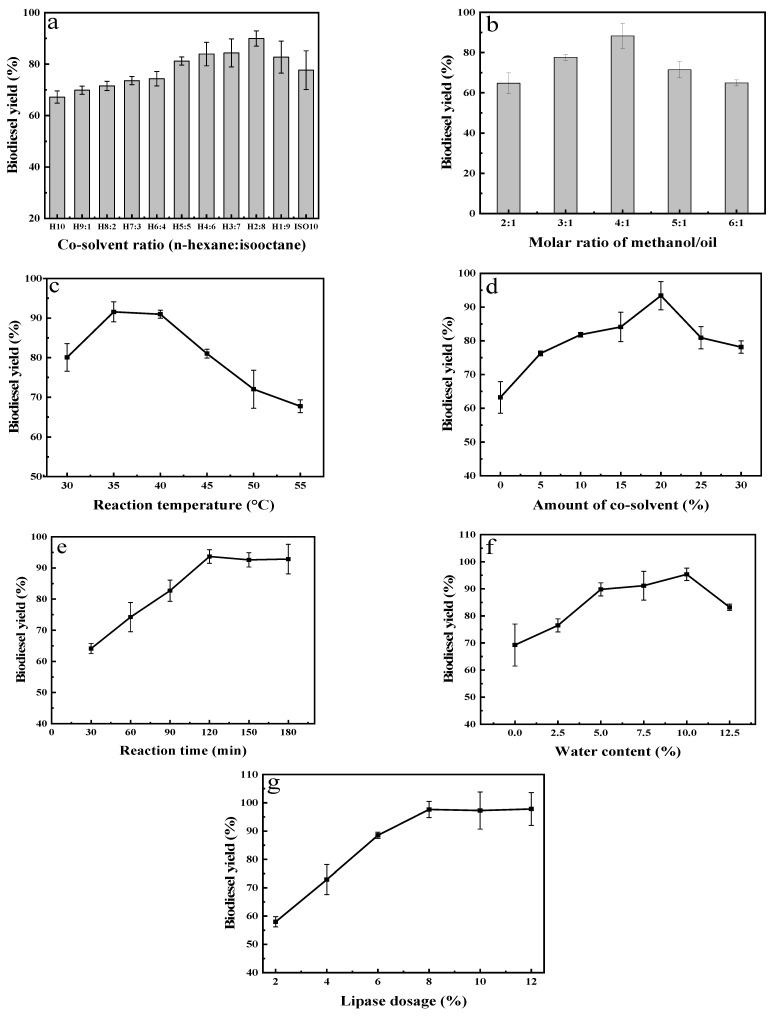
Effect of different transesterification parameters on the yield of biodiesel (%): (**a**) co-solvent ratio; (**b**) molar ratio of methanol to oil; (**c**) reaction temperature; (**d**) amount of co-solvent; (**e**) reaction time; (**f**) water content; (**g**) lipase dosage. The optimal conditions were the following: 1:4 n-hexane to isooctane ratio; 4:1 molar ratio of methanol to oil; 35 ± 1 °C reaction temperature; 20% co-solvent; 120 min reaction time; 8% lipase dosage.

Several studies have been performed on the enzymatic transesterification of methanol and ethanol with oil for producing biodiesel, and they found that these alcohols were toxic to the immobilized enzymes [38]. Thus, using organic solvents in the reaction system could decrease the inhibition of lipase by alcohol and improve lipase’s catalytic activity [6]. Hence, different ratios of co-solvents (n-hexane and isooctane) were employed in the WCO transesterification process, for dissolving WCO and methanol, and eliminating methanol’s toxicity on co-immobilized lipases (Figure 9a). As can be seen, the co-solvent reaction systems produced higher biodiesel yields than single organic solvents, and the isooctane catalytic system performed better than the n-hexane catalytic system. In addition, the results showed that the biodiesel yields in the co-solvent systems increased gradually with an increased volume ratio of isooctane. The maximum production was observed in the co-solvent system with 80% isooctane and 20% n-hexane (*v*/*v* ratio) (89.97%). These results were also proved by other studies [18,46], which used co-solvents reaction systems in the transesterification reactions, and they found that the co-solvents reaction systems were better than pure organic systems for biodiesel production. A possible explanation for this might be the improved miscibility of WCO, methanol, and biodiesel, as well as the good solubility of methanol and glycerol in the appropriate ratio of n-hexane and isooctane co-solvent. Therefore, the harmful effects of methanol and glycerol on the activity and stability of co-lipases could be significantly weakened [47]. After optimizing different reaction settings, a co-solvent solution composed of 20% n-hexane and 80% isooctane was chosen as the ideal reaction medium for biodiesel synthesis.

Alcohols play two roles in enzymatic transesterification reactions. They act as a substrate in these reactions, enhance the rate of the reactions, and push them toward biodiesel synthesis [36]. Furthermore, they are toxic to proteins, if present in high concentrations, and affect the activity and stability of CIL-mMWCNTs@4-arm-PEG-NH_2_ by attaching it to the insoluble methanol in the transesterification medium, resulting in a decline in biodiesel production [48,49]. Hence, the co-immobilized lipases were optimized at various molar ratios of methanol to oil, to protect them from methanol’s negative impact and improve the yield of biodiesel. Based on the results (Figure 7b), there is an increase in biodiesel yield when the methanol to WCO molar ratio is gradually increased from 2:1–4:1, but beyond 4:1 ratio, the yield started to decrease. Thus, the optimal methanol to WCO molar ratio was 4:1 with 88.28% of biodiesel yield. These results could be attributed to the inactivation of co-immobilized lipases with specific amounts of methanol [50]. Liu et al. [51] applied BCL-NKA to produce biodiesel and obtained the maximum conversion rate when the molar ratio of methanol to oil was 4:1 in both free-solvent and isooctane systems. Poppe et al. [52] achieved the highest yield of biodiesel (70%) with 9:1 molar ratio of ethanol to waste oil.

Enzymatic transesterification is an endothermic reaction, and each enzyme has a unique optimal temperature for catalytic activity [9]. The effects of different reaction temperatures (30–55 °C) on the WCO transesterification using co-immobilized lipases were investigated (Figure 7c). The highest biodiesel yield (91.57%) was observed at a reaction temperature of 35 ± 1 °C. As the reaction temperature elevated from 35 to 40 ± 1 °C, the biodiesel production slightly declined from 91.57% to 90.98%. A further increase in reaction temperatures induced a notable decrease in the yield. However, the lowest yield of biodiesel (67.73%) was obtained at 55 ± 1 °C. This might be due to the high reaction temperature that denatured the CIL-mMWCNTs@4-arm-PEG-NH_2_, which leads to a fast reduction of its enzymatic activity. Lee et al. [53] achieved a maximum conversion rate of biodiesel at 45 °C when they applied co-immobilized CRL and ROL onto silica gel support. Shahedi et al. [14] obtained the highest biodiesel yield at 35.6 °C when they used co-immobilized *C. antarctica* B and *R. miehei* onto epoxy functionalized silica gel support. In order to prevent thermal inactivation of lipases and save energy, lipase-catalyzed transesterification is usually done at a temperature lower than that of the chemical process [54]. Hence, the ideal temperature of the transesterification reaction catalyzed by co-immobilized lipases was set at 35 ± 1 °C.

The amounts of organic solvents supplied to enzyme-catalyzed transesterification reactions is essential. The effects of co-solvent amounts, ranging from 0% to 30%, on the yield of biodiesel were measured. Figure 9d shows that the yield of biodiesel progressively increased from 76.27% to 93.39% when 5% and 20% of co-solvent were introduced into the reaction system, respectively. Over 20%, biodiesel production gradually declined. Simultaneously, the results indicated that the biodiesel yield produced in the organic co-solvent systems was greater than that produced in the solvent-free system, which was consistent with the previous results reported by Adnan et al. [49]. This could be explained as the methanol’s deactivation of co-immobilized lipases was significantly minimized using an appropriate amount of co-solvent. The interaction between the amino acids, residual in lipase lid, and the molecules of the co-solvent, keeps the co-immobilized lipases in an open form, increasing the biological activity [55]. Consequently, the ideal addition amount of co-solvent was set at 20% for further investigation.

Generally, a long reaction time helps to complete the enzymatic transesterification reactions. The effects of different reaction times (30–180 min) on the biodiesel yield from WCO were evaluated (Figure 9e). As shown, the biodiesel yield was increased rapidly with increased reaction time from 30 to 180 min. The maximum product yield (93.69%) was obtained at 120 min. In contrast, when the reaction time exceeded 120 min, the biodiesel yield did not elevate significantly, indicating that the lack of substrate and the transesterification reaction reached its equilibrium. Our results revealed that the performance of CIL-mMWCNTs@4-arm-PEG-NH_2_ was significantly superior to that of previous studies, which required a longer reaction time to obtain a high yield of biodiesel when single or mixed immobilized lipases were used [6,18,29,52,56,57]. Similar results to the current findings were reported by Liu et al. [58], who found that the ultrasound-assisted biocatalyst process improved mass transfer rate and significantly reduced the time desired for producing biodiesel. Tupufia et al. [20] found that the highest biodiesel yield (92%) was achieved from the coconut oil transesterification catalyzed by Novozyme-435 after 3 h, with the assistance of ultrasonic process, compared to 80% conversion which was observed at 50 h under conventional reaction conditions at 350 rpm stirring speed. It can be explained that the slow reaction rate could be due to insufficient mixing between alcohol and oil, resulting in partial miscibility with each other. Ultrasonic transesterification is associated with cavitation, which provides the necessary activation energy for the reaction; thereby, increasing its speed rate. Additionally, the shock wave produced by the cavitation bubbles aids in the faster spread of substrates towards the enzyme. It can also reduce the mass transfer resistance, which frequently hinders the progress of enzymatic reactions [59]. In the current study, a combination of co-immobilized lipase with different site specificities and ultrasound techniques provides the appropriate reaction time (120 min) for desirable biodiesel yield. This rapid reaction time to synthesize biodiesel using WCO seems to possess significant potential in industrial applications.

In transesterification reactions catalyzed by enzymes, the water content also plays a crucial role. It significantly impacts lipase’s activity and stability [38,50]. Therefore, sufficient water is required when using organic solvents to keep the highest lipase activity [16]. As a result, the impact of different water contents ranging from 2.5% to 12.5% (wt., based on WCO) on the production of biodiesel was studied, and the results are presented in Figure 9f. The biodiesel yield in aqueous media was better than that in non-aqueous media. This result corroborated those of the previous report [38]. The maximum biodiesel yield of 95.34% was collected in 10% water, catalyzed by CIL-mMWCNTs@4-arm-PEG-NH_2_; however, the biodiesel yield decreased quickly when the water content was above 10%. The co-immobilized lipases act at the interface of the aqueous and non-aqueous phases: their activity depends on the availability of the interfacial region. Therefore, it is necessary to add adequate water to keep the activity of lipase and increase the obtainable water–oil interfacial region; thus, increasing the reaction’s efficiency. On the other hand, in the reaction mixture, increasing the water content could dilute the quantity of methanol and convert the transesterification reaction into the hydrolysis reaction [60]. According to the previous findings, the ideal water content for transesterification ranges between 0 and 12.5%, based on lipases and solvent systems. The ideal water contents were 10% for RML, 7.5% for CRL, and 5% for BCL in the n-octane, isooctane, and t-butanol systems, respectively [36]. In this study, the water content of 10% was deemed as the ideal moisture for WCO transesterification catalyzed by co-immobilized ROL and CRL lipases.

The effect of CIL-mMWCNTs@4-arm-PEG-NH_2_ dosage on the yield of biodiesel was optimized to balance the benefit and the cost of the lipase loading. As seen from Figure 9g, the yield of biodiesel progressively increased as the lipase dosage was increased from 2% to 8%. The best conversion rate was estimated to be 97.64%. There were no remarkable increases in biodiesel yields when the lipase dosage was beyond 8%. The main reason was that the excessive lipase dosage was easy to agglomerate and affected the mass transfer resistance, as well as lipases, which can be inactivated by methanol [6,61].

Briefly, 97.64% of biodiesel yield from WCO could be obtained with 8% of co-immobilized lipase dosage, under the parameter’s reaction of 4:1 methanol to oil molar ratio (methanol amount was added 3 times at intervals of 40 min), 20% co-solvent, 10% water content, at 35 ± 1 °C, and reacted for 120 min under ultrasound-assisted parameters of 40% power and 40 kHz frequency.

### 2.4. Reusability of Co-Immobilized Lipases–mMWCNTs@4-arm-PEG-NH_2_

**Figure 10:**
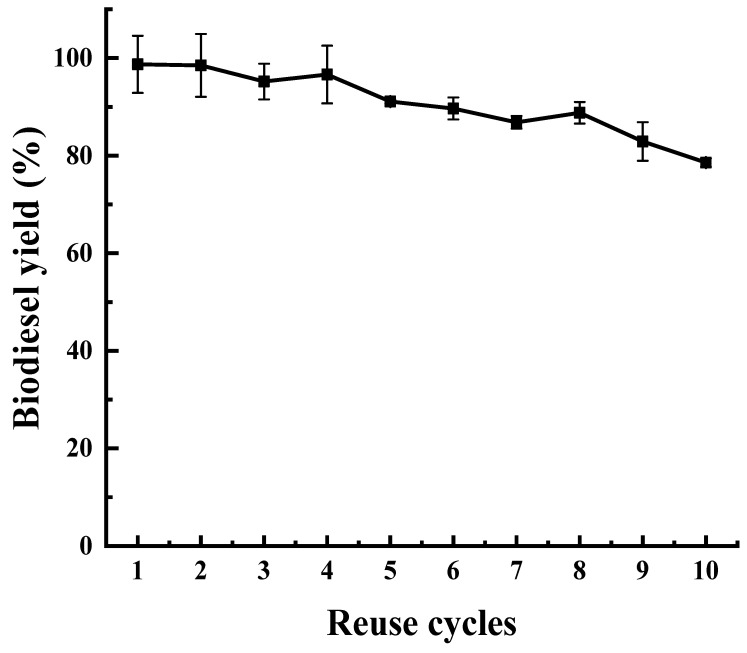
Effect of reuse of co-immobilized lipase on the yield of biodiesel (%). Reaction parameters were as follows: 4:1 molar ratio of methanol to oil (methanol was added 3 times at intervals of 40 min); 20% co-solvent; 10% water content; 8% lipase dosage; at 35 ± 1 °C; 120 min reaction time; under ultrasound-assisted parameters of 40% power and 40 kHz frequency (all % based on oil weight).

One of the goals of using co-immobilized lipases is to create a highly effective biocatalyst that is rapidly recoverable and reusable. Reusability of the immobilized enzymes is essential for their successful practical applications, because this is the key factor to reduce production costs; therefore, the application of CIL-mMWCNTs@4-arm-PEG-NH_2_ for producing biodiesel from WCO in a co-solvent system was examined to establish its reusability under the previously mentioned optimal reaction conditions. As presented in Figure 10, the co-immobilized lipase was reused 10 times and still maintained its initial activity at the first 3 batches, revealing that CRL and ROL did not readily leak from their support under processing conditions. Despite the harmful effects of methanol, co-solvent, and ultrasound waves, 78.55% of product yield was retained after 10 batches running, proving much better stability of co-immobilized lipases. It was reported that combi-immobilized lipases (*C. rugosa* and *R. miehei*) were immobilized onto a polyhydroxybutyrate support by physical adsorption. The production of biodiesel was around 30% after the 10th cycle [16]. In the process of reuse, the general reasons for the decline of biodiesel production may be attributed to the leakage of enzymes from the supports upon during the recovery and washing processes and the decrease of the immobilized enzyme activity [60]. In this study, in comparison with other immobilization methods, covalent cross-linking effectively reduced enzyme leaching and enhanced enzyme stability.

### 2.5. Comparison of CIL-mMWCNTs@4-arm-PEG-NH_2_ and other Mixtures of Immobilized Lipases

To assess the significance of this co-immobilization procedure, obtained results were compared with some previous studies that used a single and a mixture of lipases for biodiesel synthesis (Table 2). There are no published studies on the usage of co-immobilized lipases onto mMWCNTs@4-arm-PEG-NH_2_ for ultrasound-assisted biodiesel synthesis.

Table 2 shows various substrates that were converted to biodiesel using a mixture of two or three immobilized lipases and single immobilized lipases. These findings indicated that the majority of immobilized lipases required a longer reaction time for higher biodiesel production. However, CIL-mMWCNTs@4-arm-PEG-NH_2_ achieved a maximal yield of biodiesel (97.64%) in the first 120 min, exceeding the individual immobilized lipases significantly [1,36]. Moreover, CIL-mMWCNTs@4-arm-PEG-NH_2_ had a faster reaction time than all mixtures of liquid, commercial, or synthesized immobilized lipases described in Table 2 [5,14,16,18,23,26,50,52]. According to the latest study [16], WCO was employed as a feedstock for producing biodiesel catalyzed by combined immobilized lipases (*C. rugosa* and *R. miehei*), which produced 96.5% of biodiesel yield. However, the combination of *R. oryzae* and *C. rugosa* lipases in 4:1 ratio was also investigated. Both enzymes were non-immobilized and mixed in a liquid state, and the highest biodiesel yield was 98.16%. Nevertheless, the disadvantage of liquid enzymes is that they cannot be reused [26]. Additionally, only 78.3% of biodiesel yield was obtained when applying co-immobilized *R. miehei* and *C. antarctica* B lipase onto epoxy functionalized silica gel at a 1:2.5 ratio, in a t-butanol solvent system [14]. On the other hand, the immobilized lipase’s activity and reusability depend on the immobilization approaches and the reaction conditions. For this, the yield of biodiesel produced by combined immobilized *C. rugosa* and *R. miehei* lipases onto polyhydroxybutyrate by physical adsorption was reported to be 97.1% in 12 h, but this lipase could be reused 15 times with only 10% of its initial activity [50]. As compared with this study, the highest yield of biodiesel 97.64% was obtained within 120 min. Also, the number of reuse cycles of CIL-mMWCNTs@4-arm-PEG-NH_2_, under ultrasonic-assisted conditions, was greater than that of a mixture of commercial immobilized lipases (Lipozyme TL-IM, Lipozyme, RM-IM, and Novozym 435) under the same technique conditions. This compound lipase lost about 50% of its initial activity in the second time of reuse [52]. However, the biodiesel yield produced by a single immobilized *C. rugosa* lipase onto dendrimer-coated mMWCNTs by covalent bond was obtained 85.1% in 40 h, and the lipase could be reused 10 times with 58% of its initial activity [36]. Thus, CIL-mMWCNTs@4-arm-PEG-NH_2_ exhibited an excellent performance in producing biodiesel compared with other single and mixtures of liquid, immobilized, and commercial lipases, as reported so far.

**Table 2:** Comparison of transesterification reaction parameters on biodiesel yield and reuse cycle between co-immobilized ROL and CRL-mMWCNTs@4-arm-PEG-NH_2_ and other individual and mixtures of immobilized lipases.

Lipase	Mixed Lipases Ratio	Type of Lipase	Substrate	System	Operating Conditions	Biodiesel Yield (%)	Reuse Cycles and Last Yield (%)	Ref.
Novozym 435: Lipozyme TL IM	0.98:1.02	Compound commercial immobilized lipases	Stillingia oil	Co-solvent acetonitrile: t-butanol	Biocatalyst (4.32%); methanol to oil ratio (6.4:1); co-solvent (40:60%); 40 °C; 200 rpm; **12 h**	96.38	30th cycle; ≥60	[18]
*R. oryzae*: *C. rugosa*	01:01	Co-immobilized lipase onto silica gel support	Waste soybean oil	Solvent-free	Biocatalyst (20%); methanol as acyl acceptor; water content (10%); 45 °C; 250 rpm; **4 h**	97	-	[53]
*R. oryzae*: *C. rugosa*	04:01	Combined lipase in liquid form	Rapeseed oil deodorizer distillates	Solvent-free	Biocatalyst (200U/g); methanol to oil ratio (167μL); water content (46); 34 °C; 200 rpm; **6 h**	98.16	-	[26]
*C. antarctica* B: *Pseudomonas cepacia*	01:03	Combi-immobilized onto amino functionalized SBA-15 support	Lipids of oleagi-nous microalgae (Isochrysis galbana)	Solvent-free	Biocatalyst (15mg); ethanol as acyl acceptor; 50 °C; 300 rpm; **24 h**	97.2	10th cycle; 70	[5]
*C. rugosa:* *R. miehei*	01:01	Combi-immobilized onto polyhydroxybutyrate support	WCO	Solvent-free	Biocatalyst (1%); methanol to oil ratio (6:1); water content (5%); 45 °C; 250 rpm; **24 h**	96.5	10th cycle; ≥30	[16]
*C. antarctica B:* *R.miehei*	2.5:01	Co-immobilized lipase onto epoxy functionalized silica gel support	Palm oil	T-butanol	t-butanol (39.9%); methanol to oil ratio (5.9); 35.6 °C; **33.5 h**	78.3	-	[14]
*C. rugosa:* *R. miehei*	1.5:01	Combi-immobilized onto polyhydroxybutyrate support	Chicken waste oil	Solvent-free	Biocatalyst (2.5%); methanol to oil ratio (6:1); water content (5%); 40 °C; 200 rpm; **12 h**	97.1	15th cycle; 10	[50]
Lipozyme TL-IM: Lipozyme RM-IM: Novozym *435*	1.6:1:1.4	Combi-commercial immobilized lipases	Waste oil	Solvent-free	Biocatalyst (25%); ethanol/oil ratio (9:1); 40 °C; **18 h** under ultrasound-assisted reaction	70	2nd cycle; lost 50% of its initial activity	[52]
*C. rugosa*		Individual immobilized lipase on dendrimer -coated mMWCNTs	Soybean oil	Isooctane	Biocatalyst (10%); methanol to oil ratio (4:1); water content (7.5%); 40 °C; 200 rpm; **40 h**	85.1	10th cycle; 58%	[36]
*R. oryzae*		Individual immobilized lipase in microcapsules	Soybean oil	-	Biocatalyst (1g of lipase loading; ethanol/oil ratio (4:1); water content (30%); 45°C; 200 rpm; **48 h**	82.86	10th cycle; 42.98%	[1]
*R. oryzae:* *C. rugosa*	04:01	Co-immobilized lipases onto mMWCNTs@4-arm-PEG-NH_2_	WCO	Co-solvent n-hexane: isooctane	Biocatalyst (8%); methanol to oil ratio (4:1); co-solvent (20%); water content (10%); 35 °C; **120 min**; under ultrasound-assisted reaction	97.64	10th cycle; 78.55	This study

-: non data available.

## 3. Materials and Methodology

### 3.1. Materials

*Rhizopus oryzae* lipase (ROL) and *Candida rugosa* lipase (CRL) powders were commercially purchased from Sigma-Aldrich (Shanghai, China). Multi-walled carbon nanotubes (MWCNTs, 60–100 nm in diameter, with >95% purity) were bought from Nanotech Port Co., Ltd. (Shenzhen, China). Four-arm polyethylene glycol amine (4-arm-PEG-NH_2_, 10K) was obtained from Shanghai Zhenzhong Biotech Co., Ltd. (Shanghai, China). Lauric acid, N-ethyl-N-(3-(dimethylamino) propyl) carbodiimide hydrochloride (EDC), glutaraldehyde (GA), and other chemicals were procured from Sinopharm Chemical Reagent Co., Ltd. (Shanghai, China). Waste cooking oil (WCO) was thankfully donated by ZTE Agrivalley Co., Ltd. (Hubei, China), and its composition is shown in Figure S1 and Table S1.

### 3.2. Methodology

#### 3.2.1. Synthesis of mMWCNTs@4-arm-PEG-NH_2_ Nanocomposites

In this step, the MWCNTs were firstly oxidized by HNO_3_/H_2_SO_4_ conc. in a volume ratio of 1:3 for 4 h to obtain carboxyl-grafted MWCNTs, via a method previously reported by Mubarak et al. [37]. Then, the oxidized MWCNTs were filled by magnetic nanoparticles (Fe_3_O_4_), using a method mentioned by Fan et al. [41]. The final product was magnetic MWCNTs-COOH (mMWCNTs).

Subsequently, the surfaces of the mMWCNTs were activated using 4-arm-PEG-NH_2_ in a manner similar to that described by Rong et al. [33], with a slight modification. To carry out this reaction, 200 mg of mMWCNTs and 20 mL (5 mg/mL) of 4-arm-PEG-NH_2_ were added to a reaction flask containing 200 mL deionized water. The reaction mixture was sonicated for 30 min. Next, EDC (40 mg) was added to the reaction mixture and stirred at 28 °C for 2 h. After that, EDC (52 mg) was introduced into the above reaction and then continuously stirred for 12 h at 28 °C. Following the reaction time, the final product was magnetically separated, rinsed with DI water until neutral pH, and finally dried via a vacuum freeze drier. The product obtained was identified as mMWCNTs@4-arm-PEG-NH_2_, and was finally dried via a vacuum freeze drier. The product obtained was identified as mMWCNTs@4-arm-PEG-NH_2_.

#### 3.2.2. Co-Immobilization of ROL and CRL onto mMWCNTs@4-arm-PEG-NH_2_

Both ROL and CRL lipases were covalently co-immobilized onto mMWCNTs@4-arm-PEG-NH_2_ using previously described methods [22,41] with minor modifications. Firstly, an appropriate amount of magnetic material was added to a 25% glutaraldehyde solution (GA) and shaken using a water bath shaker at reaction temperature 50 °C and 200 rpm for 6 h. Then, the sample was separated using magnetic separation, rinsed with ionized water to remove excess GA, and then dried in a vacuum freeze drier. The resultant sample was detected as mMWCNTs@4-arm-PEG-NH_2_-GA. Next, a specific amount of ROL:CRL ratio (protein content 0.86 mg in all ratios) was dispersed in a 5mL phosphate buffer solution (PBS; 0.05M). Subsequently, 0.1 g mMWCNTs@4-arm-PEG-NH_2_-GA was introduced into the lipase solution, and then the solution was ultrasonically dispersed for a few seconds. Then, the reaction solution was stirred in a shaker device with 200 rpm of stirring speed at a specific immobilization temperature for a certain time. After coupling time, the co-immobilized lipase was separated using a magnet and washed with a new PBS for removing unbound lipases. Finally, the co-immobilized lipases (CIL-mMWCNTs@4-arm-PEG-NH_2_) were dried via the lyophilization method and kept at 4 °C until the subsequent usage.

In the meantime, the quantities of immobilized ROL and CRL on the support were calculated by determining the concentration of protein using bovine serum albumin (BSA), according to Bradford’s approach [62]. Single-factor optimization, including ROL lipase ratio (0.0−1 based on all lipases), glutaraldehyde concentration (2.5–12.5 wt.%), pH value (5.5–8.0), immobilization temperature (25–50 °C), and immobilization time (1–4 h) were investigated to enhance the immobilization efficiency and specific activity of the co-immobilized lipases. For comparison purposes, ROL and CRL enzymes were immobilized alone onto mMWCNTs@4-arm-PEG-NH_2_ by following the same previous steps and comparing the results with co-immobilized lipases.

#### 3.2.3. Assay of Lipase Activity and Thermal Stability

Both activities of the immobilized and free lipases were determined using a previously published procedure by Talukder et al. [63]. The esterification reaction of 1-dodecanol with lauric acid was used to estimate them. The protein content of each free and immobilized lipase was 0.26 mg. The percentages of immobilization efficiency and specific activity (U/mg protein) were measured according to the previous equations [9].

(1) $$\mathrm{Immobilization}\;\mathrm{efficiency}\;\left(\%\right)=\frac{\mathrm{immobilized}\;\mathrm{protein}}{\;\mathrm{total}\;\mathrm{loading}\;\mathrm{protein}\;}\;\times 100$$

(2) $$\mathrm{Specific}\;\mathrm{activity}\;\left(U/\mathrm{mg}\;\mathrm{protein}\;\right)=\frac{\;\mathrm{initial}\;\mathrm{activity}\;\left(U\right)}{\;\mathrm{protein}\;\mathrm{content}\;\mathrm{of}\;\mathrm{lipase}\;\left(\mathrm{mg}\right)}$$

In addition, the thermal stabilities of co-immobilized lipases, free ROL, and free CRL were respectively investigated. All lipases were incubated at varying temperatures from 30 to 70 °C for 1 h to determine their residual activity.

#### 3.2.4. Characterization

The surface chemistry of the synthesized samples was performed by X-ray photoelectron spectroscopy (XPS) using a Kratos Axis Ultra instrument, equipped with a monochromated Al Kα X-ray source (Shimadzu Kratos Co., AXIS-ULTRA DLD-600W, Kyoto, Japan). The morphological properties of the fabricated nanotubes were achieved using a transmission electron microscope (TEM, H-7000FA, Hitachi, Tokyo, Japan) and a scanning electron microscope (FSEM, Sirion 200 FEG, FEI Co., Eindhoven, The Netherlands). Patterns of powder X-ray diffraction (P-XRD) were performed with an empyrean X-ray diffractometer (PAN analytical B.V., Almelo, The Netherlands) at room temperature. The spectra of FT-IR were obtained from Fourier transform infrared spectroscopy (Bruker, VERTEX 70, Karlsruhe, Germany) [41]. The vibrating sample magnetometer (VSM) was used to determine the magnetic characteristics of the magnetic samples (Model 7404, Lake Shore, Westervilli, USA). The surface area of crude MWCNTs, oxidized MWCNTs, mMWCNTs, and mMWCNTs@4-arm-PEG-NH_2_ were determined through Brunauer–Emmett–Teller (BET) model using the ASAP^®^ 2420 surface area and porosimetry analyzer (Micromeritics Instrument (Shanghai), Norcross, GA, USA) [64]. To observe the ROL–CRL-mMWCNTs@4-arm-PEG-NH_2_, confocal laser scanning microscopy was used, (CLSM) with a SIM scanner (Olympus FV1000 Co., Tokyo, Japan). ROL and CRL were labeled with fluorescein isothiocyanate (FITC) and Rhodamine B, respectively, and the details of this process were previously described [1].

#### 3.2.5. Enzymatic Transesterification and GC Analysis for Biodiesel Production

Biodiesel production and GC analysis protocols have been slightly modified from previous procedures [6,26,38]. An ultrasound-assisted procedure was applied to monitor the reaction. The reaction mixture, containing (0.495 g) of WCO and different dosage percentages of co-solvent (n-hexane and isooctane), methanol, water, and (CIL-mMWCNTs@4-arm-PEG-NH_2_), were placed in a capped flask (50 mL) in an ultrasonic bath and set to 40 kHz frequency and 40% power. The percentages of each dosage were calculated based on WCO weight. After a specified reaction time, 400 μL of the mixture was obtained and then subjected to centrifuge at 12,000× *g* for 5 min to collect the supernatant. Next, the supernatant sample (10 μL) was mixed with n-hexane (290 μL) and heptadecanoic acid methyl ester (300 μL, 1.0 mg/mL). Then, 2.0 μL of the sample mentioned above was injected into a gas chromatograph (GC-9790, FULI). The column of this analytical instrument was an Agilent J&W HP-INNOWAX (19091N-133I) capillary column. Its temperature was increased from 180 °C to 230 °C at the ratio of 3 °C per min and was maintained for 2–3 min. Temperatures of detector and injector were saved at 280 °C and 240 °C, respectively. The yield of biodiesel (%) was estimated by the procedure described previously [26].

#### 3.2.6. Reusability of Co-Immobilized Lipases–mMWCNTs@4-arm-PEG-NH_2_

The reusability of co-immobilized lipases was measured. Following each batch reaction, the CIL-mMWCNTs@4-arm-PEG-NH_2_ was magnetically recovered and washed with a cold co-solvent. It was then lyophilized and reintroduced into the subsequent batch of the new reaction solution. The stability of CIL-mMWCNTs@4-arm-PEG-NH_2_ was examined after ten continuous batches running.

All experiments were done in three replicates, and the mean value ± standard deviation was used to express the analytical data.

## 4. Limitation of the Study and Future Research

This study established that a novel mMWCNTs@4-arm-PEG-NH_2_ support is suitable for enzyme co-immobilization and that the created CIL-mMWCNTs@4-arm-PEG-NH_2_ is a potential nano-biocatalyst for biocatalytic processes. Unlike a layer-by-layer co-immobilization method, in this study, we used a random co-immobilization method, where the ROL and CRL were co-immobilized simultaneously on the same support. This makes it difficult to determine the amount of each lipase on the carrier, which is considered as a limitation for this study. We also believe that, if we had used another nonspecific lipase instead of CRL, the sonication time needed for efficient biodiesel synthesis would have been reduced. Thus, future studies should address the limitations mentioned above, including the concurrent immobilization of two other lipases with different specificity onto mMWCNTs@4-arm-PEG-NH_2_, using a layered co-immobilization method to increase biodiesel production in shorter time, which will confirm the advantages of using this carrier for catalytic purposes, and its potential for various industrial applications.

## 5. Conclusions

This study presents mMWCNTs@4-arm-PEG-NH_2_, a novel composite of magnetic nanotubes with four-arm polyethylene glycol amine, which has been successfully synthesized and employed to co-immobilize ROL and CRL enzymes. The synthesized composite exhibited notable performance in co-immobilizing lipases as its esterification activity was 34.5-fold higher than that of free ROL, and its thermal stability was significantly enhanced. Under the assistance of ultrasound, CIL-mMWCNTs@4arm-PEG-NH_2_ was used as a biocatalyst to prepare biodiesel from WCO, and the highest biodiesel yield reached 97.64% within 120 min. Furthermore, in the batches experiment, CIL-mMWCNTs@4arm-PEG-NH_2_ showed better operational stability, and its activity could remain 78.55% after the 10th batch running. Thus, the newly developed procedure of co-immobilized ROL–CRL-mMWCNTs@4-arm-PEG-NH_2_ and the ultrasonic technique indicate their excellent potential in industrial applications.

## Figures and Tables

**Table 1:** Element information of MWCNTs after treatment.

Items	Peak	Position BE (eV)	Atomic Mass %	Atomic Content %	Mass Content %
MWCNTs	O_1s_	532.500	15.999	0.75	1.00
	C_1s_	284.300	12.011	99.25	99.00
MWCNTs-COOH	O_1s_	531.750	15.999	11.03	14.17
	C_1s_	284.300	12.011	88.97	85.83
mMWCNTs	Fe_2p_	711.050	55.846	3.80	14.95
	O_1s_	531.550	15.999	12.65	14.27
	C_1s_	284.350	12.011	83.55	70.78
mMWCNTs@4-arm-PEG-NH_2_	Fe_2p_	711.40	55.846	2.77	11.02
	O_1s_	531.25	15.999	19.71	22.46
	N_1s_	399.70	14.007	1.4	1.4
	C_1s_	284.350	12.011	76.12	65.12
Lipase-mMWCNTs@4-arm-PEG-NH_2_	Fe_2p_	711.450	55.846	0.85	3.65
	O_1s_	532.550	15.999	16.00	19.61
	N_1s_	400.300	14.007	1.44	1.54
	C_1s_	284.400	12.011	81.70	75.17
	S_2p_	165.150	32.065	0.010	0.020

## Data Availability

Not applicable.

## Supplementary Materials

The following are available online at https://www.mdpi.com/article/10.3390/ijms222111956/s1.

## References

[1] Su F., Li G., Fan Y., Yan Y. (2016). Enhanced performance of lipase via microcapsulation and its application in biodiesel preparation. Sci. Rep..

[2] Aarthy M., Saravanan P., Gowthaman M.K., Rose C., Kamini N.R. (2014). Enzymatic transesterification for production of biodiesel using yeast lipases: An overview. Chem. Eng. Res. Des..

[3] Cavalcante F.T.T., Neto F.S., Rafael de Aguiar Falcão I., Erick da Silva Souza J., de Moura Junior L.S., da Silva Sousa P., Rocha T.G., de Sousa I.G., de Lima Gomes P.H., de Souza M.C.M. (2021). Opportunities for improving biodiesel production via lipase catalysis. Fuel.

[4] Bhan C., Singh J. (2020). Role of microbial lipases in transesterification process for biodiesel production. Environ. Sustain..

[5] Sánchez-Bayo A., Morales V., Rodríguez R., Vicente G., Bautista L. (2019). Biodiesel production (FAEEs) by heterogeneous combi-lipase biocatalysts using wet extracted lipids from Microalgae. Catalysts.

[6] Wang J., Li K., He Y., Wang Y., Han X., Yan Y. (2019). Enhanced performance of lipase immobilized onto Co^2+^-chelated magnetic nanoparticles and its application in biodiesel production. Fuel.

[7] Faizollahzadeh Ardabili S., Najafi B., Alizamir M., Mosavi A., Shamshirband S., Rabczuk T. (2018). Using SVM-RSM and ELM-RSM approaches for optimizing the production process of methyl and ethyl esters. Energies.

[8] Najafi B., Faizollahzadeh Ardabili S., Shamshirband S., Chau K.-W., Rabczuk T. (2018). Application of ANNs, ANFIS and RSM to estimating and optimizing the parameters that affect the yield and cost of biodiesel production. Eng. Appl. Comput. Fluid Mech..

[9] Adnan M., Li K., Wang J., Xu L., Yan Y. (2018). Hierarchical ZIF-8 toward immobilizing *Burkholderia cepacia* lipase for application in biodiesel preparation. Int. J. Mol. Sci..

[10] Quayson E., Amoah J., Hama S., Kondo A., Ogino C. (2020). Immobilized lipases for biodiesel production: Current and future greening opportunities. Renew. Sustain. Energy Rev..

[11] Ribeiro B.D., de Castro A.M., Coelho M.A., Freire D.M. (2011). Production and use of lipases in bioenergy: A review from the feedstocks to biodiesel production. Enzym. Res..

[12] Su F., Li G.-L., Fan Y.-L., Yan Y.-J. (2015). Enhancing biodiesel production via a synergic effect between immobilized *Rhizopus oryzae* lipase and Novozym 435. Fuel Process. Technol..

[13] Shahedi M., Habibi Z., Yousefi M., Brask J., Mohammadi M. (2021). Improvement of biodiesel production from palm oil by co-immobilization of *Thermomyces lanuginosa* lipase and *Candida antarctica* lipase B: Optimization using response surface methodology. Int. J. Biol. Macromol..

[14] Shahedi M., Yousefi M., Habibi Z., Mohammadi M., As’habi M.A. (2019). Co-immobilization of *Rhizomucor miehei* lipase and *Candida antarctica* lipase B and optimization of biocatalytic biodiesel production from palm oil using response surface methodology. Renew. Energy.

[15] Lee J.H., Kim S.B., Kang S.W., Song Y.S., Park C., Han S.O., Kim S.W. (2011). Biodiesel production by a mixture of *Candida rugosa* and *Rhizopus oryzae* lipases using a supercritical carbon dioxide process. Bioresour. Technol..

[16] Binhayeeding N., Klomklao S., Prasertsan P., Sangkharak K. (2020). Improvement of biodiesel production using waste cooking oil and applying single and mixed immobilised lipases on polyhydroxyalkanoate. Renew. Energy.

[17] Alves J.S., Vieira N.S., Cunha A.S., Silva A.M., Záchia Ayub M.A., Fernandez-Lafuente R., Rodrigues R.C. (2014). Combi-lipase for heterogeneous substrates: A new approach for hydrolysis of soybean oil using mixtures of biocatalysts. RSC. Adv..

[18] Li Q., Zheng J., Yan Y. (2010). Biodiesel preparation catalyzed by compound-lipase in co-solvent. Fuel Process. Technol..

[19] Subhedar P.B., Botelho C., Ribeiro A., Castro R., Pereira M.A., Gogate P.R., Cavaco-Paulo A. (2015). Ultrasound intensification suppresses the need of methanol excess during the biodiesel production with Lipozyme TL-IM. Ultrason. Sonochem..

[20] Tupufia S.C., Jeon Y.J., Marquis C., Adesina A.A., Rogers P.L. (2013). Enzymatic conversion of coconut oil for biodiesel production. Fuel Process. Technol..

[21] Freitas V.O.D., Matte C.R., Poppe J.K., Rodrigues R.C., Ayub M.A.Z. (2019). Ultrasound-assisted transesterification of soybean oil using combi-lipase biocatalysts. Braz. J. Chem. Eng..

[22] Lee J.H., Kim S.B., Yoo H.Y., Lee J.H., Han S.O., Park C., Kim S.W. (2013). Co-immobilization of *Candida rugosa* and *Rhyzopus oryzae* lipases and biodiesel production. Korean J. Chem. Eng..

[23] Lee J.H., Lee J.H., Kim D.S., Yoo H.Y., Park C., Kim S.W. (2019). Biodiesel production by lipases co-immobilized on the functionalized activated carbon. Bioresour. Technol. Rep..

[24] Arana-Pena S., Rios N.S., Mendez-Sanchez C., Lokha Y., Carballares D., Goncalves L.R.B., Fernandez-Lafuente R. (2020). Coimmobilization of different lipases: Simple layer by layer enzyme spatial ordering. Int. J. Biol. Macromol..

[25] Toro E.C., Rodríguez D.F., Morales N., García L.M., Godoy C.A. (2019). Novel combi-lipase systems for fatty acid ethyl esters production. Catalysts.

[26] Zeng L., He Y., Jiao L., Li K., Yan Y. (2017). Preparation of biodiesel with liquid synergetic lipases from rapeseed oil deodorizer distillate. Appl. Biochem. Biotechnol..

[27] Yang X., Zhang Y., Pang H., Yuan S., Wang X., Hu Z., Zhou Q., He Y., Yan Y., Xu L. (2021). Codisplay of *Rhizopus oryzae* and *Candida rugosa* lipases for biodiesel production. Catalysts.

[28] Rafiee F., Rezaee M. (2021). Different strategies for the lipase immobilization on the chitosan based supports and their applications. Int. J. Biol. Macromol..

[29] Fan Y., Wu G., Su F., Li K., Xu L., Han X., Yan Y. (2016). Lipase oriented-immobilized on dendrimer-coated magnetic multi-walled carbon nanotubes toward catalyzing biodiesel production from waste vegetable oil. Fuel.

[30] Wang J., Li K., He Y., Wang Y., Yan J., Xu L., Han X., Yan Y. (2020). Lipase immobilized on a novel rigid-flexible dendrimer-grafted hierarchically porous magnetic microspheres for effective resolution of (R,S)-1-phenylethanol. ACS Appl. Mater. Interfaces.

[31] Xu J., Sheng Z., Wang X., Liu X., Xia J., Xiong P., He B. (2016). Enhancement in ionic liquid tolerance of cellulase immobilized on PEGylated graphene oxide nanosheets: Application in saccharification of lignocellulose. Bioresour. Technol..

[32] Xie X., Luo P., Han J., Chen T., Wang Y., Cai Y., Liu Q. (2019). Horseradish peroxidase immobilized on the magnetic composite microspheres for high catalytic ability and operational stability. Enzym. Microb. Technol..

[33] Rong J., Zhou Z., Wang Y., Han J., Li C., Zhang W., Ni L. (2019). Immobilization of horseradish peroxidase on multi-armed magnetic graphene oxide composite: Improvement of loading amount and catalytic activity. Food Technol. Biotechnol..

[34] Han J., Wang L., Wang Y., Dong J., Tang X., Ni L., Wang L. (2018). Preparation and characterization of Fe_3_O_4_-NH_2_@4-arm-PEG-NH_2_, a novel magnetic four-arm polymer-nanoparticle composite for cellulase immobilization. Biochem. Eng. J..

[35] Zhong K., Du X., Li J. (2014). Progress in polyethylene glycol modified chitosan as drug delivery carriers. Chemistery.

[36] Fan Y., Ke C., Su F., Li K., Yan Y. (2017). Various types of lipases immobilized on dendrimer-functionalized magnetic nanocomposite and application in biodiesel preparation. Energy Fuels.

[37] Mubarak N.M., Wong J.R., Tan K.W., Sahu J.N., Abdullah E.C., Jayakumar N.S., Ganesan P. (2014). Immobilization of cellulase enzyme on functionalized multiwall carbon nanotubes. J. Mol. Catal. B Enzym..

[38] Li K., Fan Y.L., He Y.J., Zeng L.P., Han X.T., Yan Y.J. (2017). *Burkholderia cepacia* lipase immobilized on heterofunctional magnetic nanoparticles and its application in biodiesel synthesis. Sci. Rep..

[39] Anwar M.Z., Kim D.J., Kumar A., Patel S.K.S., Otari S., Mardina P., Jeong J.H., Sohn J.H., Kim J.H., Park J.T. (2017). SnO2 hollow nanotubes: A novel and efficient support matrix for enzyme immobilization. Sci. Rep..

[40] Arana-Peña S., Carballares D., Berenguer-Murcia Á., Alcántara A., Rodrigues R., Fernandez-Lafuente R. (2020). One pot use of combilipases for full modification of oils and Fats: Multifunctional and heterogeneous substrates. Catalysts.

[41] Fan Y., Su F., Li K., Ke C., Yan Y. (2017). Carbon nanotube filled with magnetic iron oxide and modified with polyamidoamine dendrimers for immobilizing lipase toward application in biodiesel production. Sci. Rep..

[42] Rehm S., Trodler P., Pleiss J. (2010). Solvent-induced lid opening in lipases: A molecular dynamics study. Protein Sci..

[43] Khan F.I., Lan D., Durrani R., Huan W., Zhao Z., Wang Y. (2017). The lid domain in lipases: Structural and functional determinant of enzymatic properties. Front. Bioeng. Biotechnol..

[44] Subhedar P.B., Gogate P.R. (2016). Ultrasound assisted intensification of biodiesel production using enzymatic interesterification. Ultrason. Sonochem..

[45] Lee D.H., Kim J.M., Shin H.Y., Kang S.W., Kim S.W. (2006). Biodiesel production using a mixture of immobilized *Rhizopus oryzae* and *Candida rugosa* lipases. Biotechnol. Bioprocess Eng..

[46] Fu B., Vasudevan P.T. (2010). Effect of solvent−co-solvent mixtures on lipase-catalyzed transesterification of canola oil. Energy Fuels.

[47] Babaki M., Yousefi M., Habibi Z., Mohammadi M., Brask J. (2015). Effect of water, organic solvent and adsorbent contents on production of biodiesel fuel from canola oil catalyzed by various lipases immobilized on epoxy-functionalized silica as low cost biocatalyst. J. Mol. Catal. B Enzym..

[48] Andrade T.A., Errico M., Christensen K.V. (2017). Influence of the reaction conditions on the enzyme catalyzed transesterification of castor oil: A possible step in biodiesel production. Bioresour. Technol..

[49] Adnan M., Li K., Xu L., Yan Y. (2018). X-Shaped ZIF-8 for immobilization *Rhizomucor miehei* lipase via encapsulation and its application toward biodiesel production. Catalyst.

[50] Sangkharak K., Mhaisawat S., Rakkan T., Paichid N., Yunu T. (2020). Utilization of mixed chicken waste for biodiesel production using single and combination of immobilized lipase as a catalyst. Biomass Convers. Biorefin..

[51] Liu T., Liu Y., Wang X., Li Q., Wang J., Yan Y. (2011). Improving catalytic performance of Burkholderia cepacia lipase immobilized on macroporous resin NKA.J. Mol. Catal. B Enzym..

[52] Poppe J.K., Matte C.R., Fernandez-Lafuente R., Rodrigues R.C., Ayub M.A.Z. (2018). Transesterification of waste frying oil and soybean oil by combi-lipases under ultrasound-assisted reactions. Appl. Biochem. Biotechnol..

[53] Lee J.H., Kim S.B., Yoo H.Y., Suh Y.J., Kang G.B., Jang W.I., Kang J., Park C., Kim S.W. (2013). Biodiesel production by enzymatic process using Jatropha oil and waste soybean oil. Biotechnol. Bioprocess Eng..

[54] Gog A., Roman M., Toşa M., Paizs C., Irimie F.D. (2012). Biodiesel production using enzymatic transesterification–Current state and perspectives. Renew. Energy.

[55] Ji Q., Xiao S., He B., Liu X. (2010). Purification and characterization of an organic solvent-tolerant lipase from *Pseudomonas aeruginosa* LX1 and its application for biodiesel production. J. Mol. Catal. B Enzym..

[56] Lopez E.N., Medina A.R., Moreno P.A.G., Cerdan L.E., Valverde L.M., Grima E.M. (2016). Biodiesel production from *Nannochloropsis gaditana* lipids through transesterification catalyzed by *Rhizopus oryzae* lipase. Bioresour. Technolo..

[57] Abahazi E., Boros Z., Poppe L. (2014). Additives enhancing the catalytic properties of lipase from *Burkholderia cepacia* immobilized on mixed-function-grafted mesoporous silica gel. Molecules.

[58] Liu Y., Chen D., Yan Y., Peng C., Xu L. (2011). Biodiesel synthesis and conformation of lipase from *Burkholderia cepacia* in room temperature ionic liquids and organic solvents. Bioresour. Technol..

[59] Gusniah A., Veny H., Hamzah F. (2018). Ultrasonic assisted enzymatic transesterification for biodiesel production. Ind. Eng. Chem. Res..

[60] You Q., Yin X., Zhao Y., Zhang Y. (2013). Biodiesel production from Jatropha oil catalyzed by immobilized *Burkholderia cepacia* lipase on modified attapulgite. Bioresour. Technol..

[61] Garmroodi M., Mohammadi M., Ramazani A., Ashjari M., Mohammadi J., Sabour B., Yousefi M. (2016). Covalent binding of hyper-activated *Rhizomucor miehei* lipase (RML) on hetero-functionalized siliceous supports. Int. J. Biol. Macromol..

[62] Bradford M.M. (1976). A rapid and sensitive method for the quantitation of microgram quantities of protein utilizing the principle of protein-dye binding. Anal. Biochem..

[63] Talukder M.M.R., Tamalampudy S., Li C.J., Yanglin L., Wu J., Kondo A., Fukuda H. (2007). An improved method of lipase preparation incorporating both solvent treatment and immobilization onto matrix. Biochem. Eng. J..

[64] Otari S.V., Patel S.K.S., Kalia V.C., Lee J.K. (2020). One-step hydrothermal synthesis of magnetic rice straw for effective lipase immobilization and its application in esterification reaction. Bioresour. Technol..

