# Supplementary material for: Co-Immobilization of Rhizopus oryzae and Candida rugosa Lipases onto mMWCNTs@4-arm-PEG-NH2—A Novel Magnetic Nanotube–Polyethylene Glycol Amine Composite—And Its Applications for Biodiesel Production
Source: Int J Mol Sci. 2021 Nov 4;22(21):11956. doi: 10.3390/ijms222111956 (PMC8584430; doi:10.3390/ijms222111956)
Supplement: Supplementary file 1 [file ijms-22-11956-s001.zip › ijms-1420586-supplementary.pdf]

Supporting Information

Co-immobilization of *Rhizopus oryzae* and *Candida rugosa* lipases onto mMWCNTs@4-arm-PEG-NH_2, a novel magnetic nanotube-polyethylene glycol amine composite, and its application for biodiesel production

Saadiah A. Abdulmalek[1,2], Kai Li[1], Jianhua Wang[1], Michael Kidane Ghide[1,3], Yunjun Yan[1,*]

[1] Key Laboratory of Molecular Biophysics of the Ministry of Education, College of Life Science and Technology, Huazhong University of Science and Technology, Wuhan, 430074, China.

[2] Department of Biology, Faculty of Science, Sana'a University, Sana'a, Yemen.

3 Department of Biology, Mainefhi College of Science, Eritrea Institute of Technology, Mainefhi, Eritrea.

*Corresponding author. Tel/Fax: +86-27-87792213;

E-mail: yanyunjun@hust.edu.cn

Results and discussion

Chemical characterization of the WCO

According to the qualitative and quantitative analysis of waste cooking oil by GC-MS, it can be seen from Table S1. the main six peaks were identified as myristic acid (C14:0, 1.05 %), palmitic acid (C16:0, 19.84 %), palmitoleic acid (C16:1, 1.25 %), stearic acid (C18:0, 6.56 %), oleic acid (C18:1, 32.24 %), linoleic acid (C18:2, 32.64 %), and linolenic acid (C18:3, 4.62 %). Thin layer chromatography analysis was also conducted (Figure S1). The standard samples from lane 1 to lane 4 were diacylglycerols (1, 2-and 1, 3-DAG), triacylglycerol (TAG), oleic acid (FFA), Soybean oil, respectively. Lane 5 was WCO. Therefore, the main component of WCO was free fatty acids, triglyceride, and a small amount of diglyceride and monoglyceride (Figure S1).

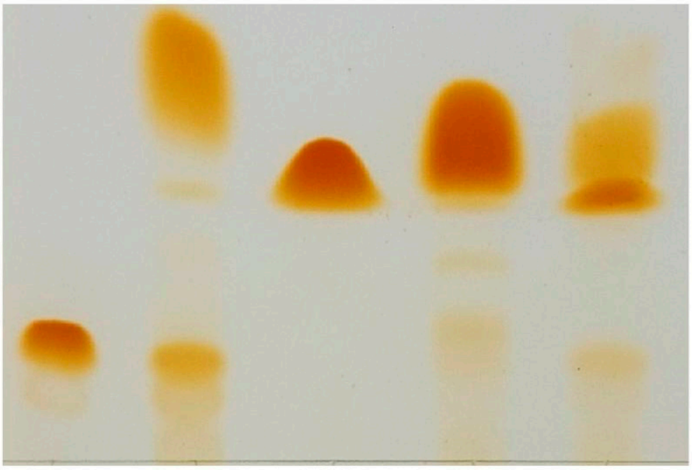

Figure S1. TLC analysis of waste cooking oil compositions.

Table S1. Analysis of fatty acid content (%) in waste cooking oil using GC-MS.

Fatty acid	Structure	Content (%)	Fatty acid	Structure	Content (%)
Myristic acid	C14:0	1.05	Oleic acid	C18:1	32.24
Palmitic acid	C16:0	19.84	Linoleic acid	C18:2	32.64
Palmitoleic acid	C16:1	1.25	Linolenic acid	C18:3	4.62
Margaric acid	C17:0	0.38	Arachidic acid	C20.0	0.36
Stearic acid	C18:0	6.56	Paullinic acid	C20:1	1.07
